# Double Allogenic Mesenchymal Stem Cells Transplantations Could Not Enhance Therapeutic Effect Compared with Single Transplantation in Systemic Lupus Erythematosus

**DOI:** 10.1155/2012/273291

**Published:** 2012-07-09

**Authors:** Dandan Wang, Kentaro Akiyama, Huayong Zhang, Takayoshi Yamaza, Xia Li, Xuebing Feng, Hong Wang, Bingzhu Hua, Bujun Liu, Huji Xu, Wanjun Chen, Songtao Shi, Lingyun Sun

**Affiliations:** ^1^Department of Rheumatology and Immunology, The Affiliated Drum Tower Hospital of Nanjing University Medical School, Nanjing 210008, China; ^2^Center for Craniofacial Molecular Biology, University of Southern California School of Dentistry, Los Angeles, CA 90033, USA; ^3^Department of Rheumatology and Immunology, Changzheng Hospital, The Second Military Medical University, Shanghai 200003, China; ^4^Mucosal Immunology Unit, Oral Infection and Immunity Branch, National Institute of Dental and Craniofacial Research, National Institutes of Health, Bethesda, MA 20892-2190, USA

## Abstract

The clinical trial of allogenic mesenchymal stem cells (MSCs) transplantation for refractory SLE patients has shown significant safety and efficacy profiles. However, the optimum frequency of the MSCs transplantation (MSCT) is unknown. This study was undertaken to observe whether double transplantations of MSCs is superior to single transplantation. Fifty-eight refractory SLE patients were enrolled in this study, in which 30 were randomly given single MSCT, and the other 28 were given double MSCT. Patients were followed up for rates of survival, disease remission, and relapse, as well as transplantation-related adverse events. SLE disease activity index (SLEDAI) and serologic features were evaluated. Our results showed that no remarkable differences between single and double allogenic MSCT were found in terms of disease remission and relapse, amelioration of disease activity, and serum indexes in an SLE clinical trial with more than one year followup. This study demonstrated that single MSCs transplantation at the dose of one million MSCs per kilogram of body weight was sufficient to induce disease remission for refractory SLE patients.

## 1. Introduction

Systemic lupus erythematosus (SLE) is an autoimmune disease characterized by multiorgan involvement and loss of tolerance against self-antigens followed by antibody production. Current treatments of severe SLE flares consist of toxic immunosuppressive drugs, most commonly cyclophosphamide (CYC), mycophenolate mofetil, and leflunomide [1]. However, the therapeutic options in cases of SLE refractory to standard treatments are indeed limited, and the disease remains potentially fatal in some patients [2].

Mesenchymal stem cells (MSCs) have potent immunosuppressive capacity, which is demonstrated by the inhibition of T lymphocytes proliferation and proinflammatory cytokines production *in vivo* and *in vitro*. MSCs, furthermore, suppress antibody production of B cells and the generation and function of antigen presenting cells [3, 4]. The immunomodulation of MSCs is for a large extent mediated by soluble factors and induced under inflammatory conditions [5]. Previous studies showed that MSCs/osteoblast linage played a critical role in maintaining the hematopoietic stem cell (HSC) niche [6, 7]. Recently, it has been demonstrated that MSCs themselves constitute an essential HSC niche component, and they are spatially associated with HSC niche *in vivo* bone marrow [8].

As the first example of efficacy, clinical trials for prevention and treatment of graft-versus-host disease (GVHD) after HSC transplantation show that MSCs can modulate allogenic immune responses and effectively treat human disease. Now these multipotential cells have been applied in various physical and immune injuries including liver cirrhosis, multiple sclerosis, and Crohn's disease [9–11]. Our previous studies also showed that allogenic bone marrow or umbilical-cord-derived MSCs transplantation is safe and effective in treating drug-resistant SLE patients [12–14]. In these pilot clinical studies, all patients received once intravenously MSCs infusion. Additionally, we found that some patients were also well responsive to another dose of MSCs after disease relapse. On the other hand, animal studies indicated that multiple MSCs transplantations could enhance clinical efficacy in lupus mice [15]. However, it is unknown whether multiple MSCs infusions are superior to single transplantation in patients, and the optimal doseage and frequency for MSCs therapy is still obscure. So in this study, we compare the efficacy between single and double transplantations of allogenic MSCs in SLE patients. The conclusion of this study can provide further potentiality of allogenic MSCs transplantation in clinical application for SLE.

## 2. Materials and Methods

### 2.1. Patients

From March 2007 through February 2010, 58 patients with SLE refractory to standard therapies were enrolled in allogenic MSCs transplantation (MSCT) trial at the Affiliated Drum Tower Hospital of Nanjing University Medical School after signing informed consent. The study was approved by the Ethics Committee at The Drum Tower Hospital of Nanjing University Medical School and registered at ClinicalTrials.gov (Identifier: NCT00698191). All enrolled patients had at least 4 of 11 American College of Rheumatology criteria for SLE [16]. The inclusion and exclusion criteria have been shown as previously [12]. The trial was conducted in compliance with current Good Clinical Practice standards and in accordance with the principles set forth under the Declaration of Helsinki (1989).

### 2.2. MSCs Purification and Identification

Bone-marrow-derived MSCs (BMMSCs) were obtained from healthy family donors after signing informed consents. Bone marrow mononuclear cells were separated by density gradient centrifugation as previously described [13, 14]. Those without appropriate bone marrow donors were infused with umbilical-cord-derived MSCs (UCMSCs). UCMSCs were prepared by the Stem Cell Center of Jiangsu Province. Fresh umbilical cords were obtained from informed and healthy mothers in local maternity hospitals after normal deliveries. The purification procedure was described as previously [12].

Criteria for release of MSCs for clinical use included presence of visible clumps, spindle-shape morphology, and absence of contamination by pathogens (as documented by aerobic and anaerobic cultures before release), as well as by virus for hepatitis B surface antigen, hepatitis B core antibody, hepatitis C virus antibody, human immunodeficiency virus antibodies I and II, cytomegalovirus IgM, and syphilis antibody (as determined by enzyme-linked immunosorbent assay [ELISA] before release), cell viability greater than 92% (as determined by trypan blue testing), and immune phenotyping proving expression of CD73, CD105, CD90, CD29 (>90%), and absence of CD45, CD34, CD14, CD79, and HLA-DR (<2%).

### 2.3. MSCs Transplantation Procedures

Randomization was conducted between once and double MSCT groups. The enrolled 58 refractory SLE patients were randomly assigned into once or double MSCT groups. Of all the patients, 30 were randomly given a single MSCs transplantation, and the other 28 patients received double allogenic MSCs transplantations, with an interval for 1 week. Before MSCT, all patients were administered CYC (10 mg per kilogram per day) intravenously on days 4, 3, and 2 to inhibit active lymphocytes. Patients received allogenic MSCs intravenously at the density of one million cells per kilogram of body weight in each transplantation.

### 2.4. Follow-Up and Outcome Characteristics

After MSCT, all patients returned for scheduled followup at 1, 3, 6, and 12 months and then yearly thereafter. Medical history, physical examination, and serologic testing were performed. Complete remission was defined as SLEDAI score < 3 and steroid requirement ≤ 10 mg/day of prednisone or its equivalent, combined with British Isles Lupus Assessment (BILAG) D scores or better in all organs but not hematological system [17, 18]. Complete remission for hematological system was defined as white blood cell count > 4,000/*μ*L, hemoglobin > 11 g/dL, platelet count > 100 × 10^9^/L, and steroid maintenance ≤ 10 mg/day of prednisone or its equivalent. Disease relapse was defined as an increase in SLEDAI score ≥ 3 from the previous visit, or experience 1 new domain with a BILAG A score or 2 new domains with a BILAG B score after a previous response [17, 18]. Transplantation-related mortality included all deaths associated with transplantation of MSCs, except those related to recurrence of underlying disease. The investigators assessed and recorded adverse events and their severity throughout the study.

After UC-MSCT, the dose of prednisone and immunosuppressive drugs was tapered when clinical efficacy was achieved for each patient. The withdrawal of prednisone and immunosuppressant was permitted if patient's condition continued to improve. No other immunosuppressant was used unless disease relapsed. If the patient underwent disease relapse, he or she will withdraw from the study and will be given higher dose of prednisone or other immunosuppressants according to disease conditions. The patient's clinical data after relapse and change of clinical regimens will not be included for analysis.

### 2.5. Statistical Analysis

Descriptive statistics was used to summarize patient characteristics. Differences in patient demographics prior transplantation were analyzed by unpaired *t-*test, Chi-Square test, or Fisher's exact test. All tests were 2 sides. Rates of overall survival, disease complete remission, relapse, and adverse event at different visit times at two groups were analyzed by a Kaplan-Meier survival curve and were statistically tested with the log-rank test. We calculated the hazard ratio (HR) and their 95% CIs using the univariate Cox proportional hazards model. Serial data were compared within and between groups by repeated measures ANOVA. All *P* value of less than 0.05 was considered as a significant difference.

## 3. Results

### 3.1. Patient Demographics and Disease Manifestations before MSCT

Fifty-eight patients with refractory SLE enrolled in this trial, and all patients underwent allogenic MSCT and were followedup for more than 12 months. The mean followup was 27 months (range from 12 to 48 months) in single transplantation group and 26 months (range from 12 to 40 months) in double transplantation group. Patients' demographics pretransplantation have been shown in Table 1. The two cohorts were balanced in gender, race, MSCs source, clinical manifestations, and disease activity prior transplantation. Medium disease duration was shorter in single than in double transplantation group (mean ± SD 60.2 ± 50.0 months versus 92.1 ± 64.3 months, *P* = 0.039).

### 3.2. Overall Survival, Disease-Free Survival, and Relapse

**Figure 1:**
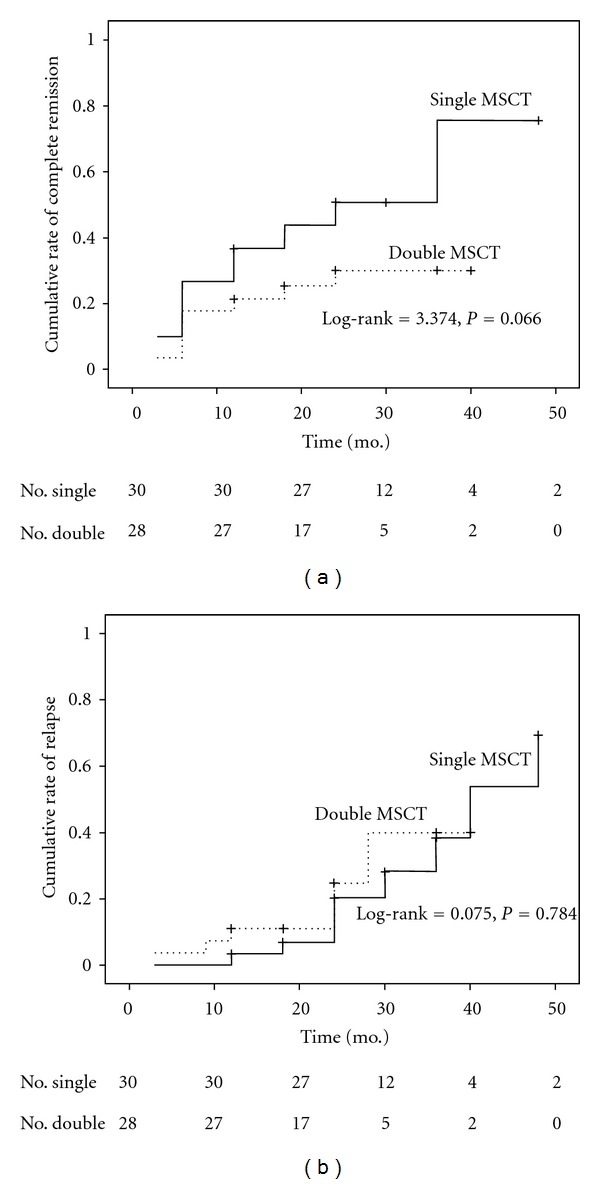
Rate of complete remission (a) and disease relapse (b) for patients with single and double MSCs transplantations, by Kaplan-Meier survival curve analysis.

With medium followup of over 24 months in both cohorts, one death was observed in double transplantation group. The survival rate was 100% for single and 96.4% for double transplantation group, respectively (log-rank = 1.071, *P* = 0.301). Rate of complete remission was 53.3% (16/30) in single transplantation group and 28.6% (8/27) in multiple transplantation group during 4-year followup by Kaplan-Meier survival curves (log-rank = 3.374, *P* = 0.066, Figure 1(a)). In a multivariable Cox regression model for complete remission, there was no difference between single and double transplantation group (HR 0.38, 95%CI 0.14–1.02; *P* = 0.060). Disease duration (HR 1.00, 95%CI 0.99–1.01, *P* = 0.290) and MSC source (HR 0.69, 95%CI 0.28–1.69, *P* = 0.420) were also not associated with complete remission. Rate of disease relapse was not statistically different in single transplantation group (8/30, 26.7%) compared to that of double transplantation group (6/27, 22.2%, log-rank = 0.075, *P* = 0.784, Figure 1(b)). The average time to relapse was 21 months (mean value, from 6 to 45 months) and 12 months (mean value, from 3 to 24 months) in single and double transplantation group, respectively. Cox regression showed that no difference in disease relapse was found between the two groups (HR 1.16, 95%CI 0.39–3.49, *P* = 0.790).

We calculated the overall percentage of disease relapse that occurred in two groups (8/30, 26.7% in single MSCT group; 6/27, 22.2% in double MSCT group). Additionally, the exact rate of disease relapse was calculated by Kaplan-Meier survival curve (Figure 1(b)), and the variable was correlated with time point when relapse occurred. In the present data, for those who had disease relapse, most relapse events occurred after 24 months followup. For example, in once MSCT group, 7 patients completed 30 months followup and had achieved a previous clinical response, in which 4 had relapse at 40, 36, 48, and 30 months, respectively (Figure 1(b), Supplementary Material available online at doi:10.1155/2012/273291).

### 3.3. Disease Activity and Serum Indexes

**Figure 2:**
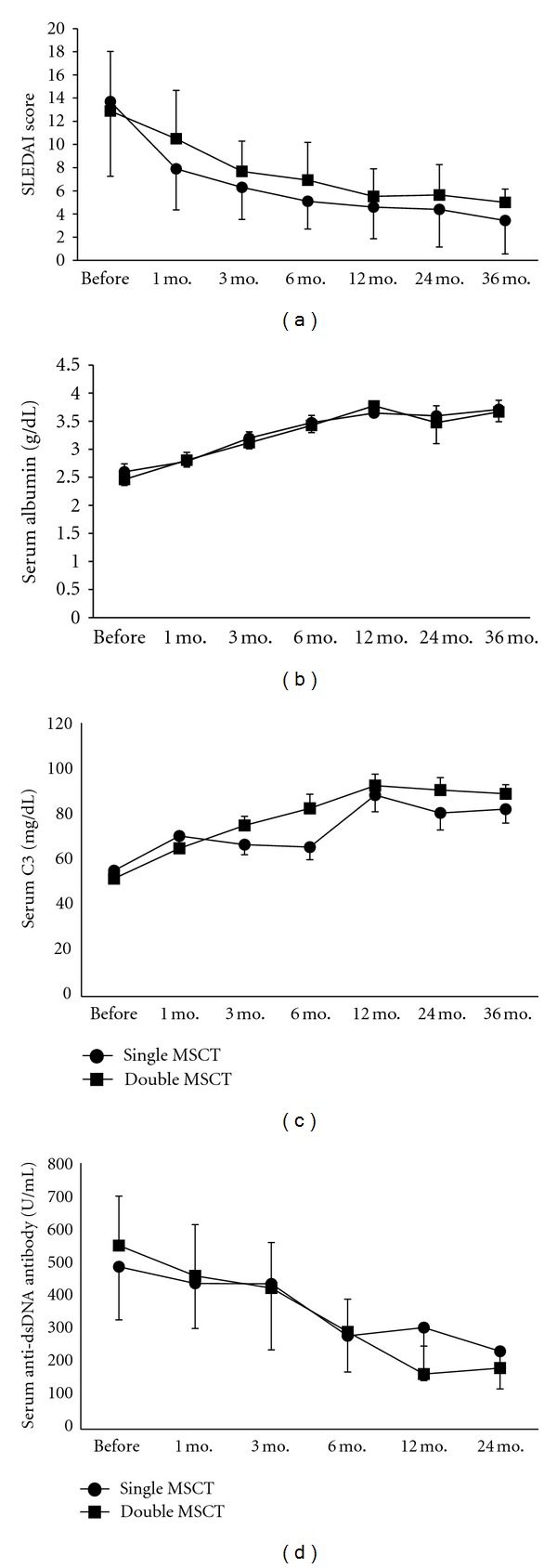
Comparisons of SLEDAI score (a), serum albumin (b), Complement 3 (C3, (c)), and anti-double-strand DNA antibody (dsDNA, (d)) for patients with single and double MSCs transplantations, by repeated measures ANOVA. Values are the mean ± SEM.

Disease activity shown by SLEDAI scores decreased significantly in both groups after allogenic MSCs transplantation by repeated measures ANOVA (*F* = 59.36, *P* < 0.001, Figure 2(a)). There was no correlation between SLEDAI decline and MSCs transplantation frequency (*F* = 3.31, *P* = 0.074). Serum albumin also significantly improved after MSCT at each group (*F* = 50.89, *P* = 0.000), and there was no difference between the two groups (*F* = 0.018, *P* = 0.896, Figure 2(b)). Serum complement 3 (C3) and anti-double-strand DNA (dsDNA) antibody similarly improved in both groups by the same analyzed methods (Figures 2(c) and 2(d)).

### 3.4. Amelioration of Renal Function and Hematologic Indexes by Allogenic MSCT

**Figure 3:**
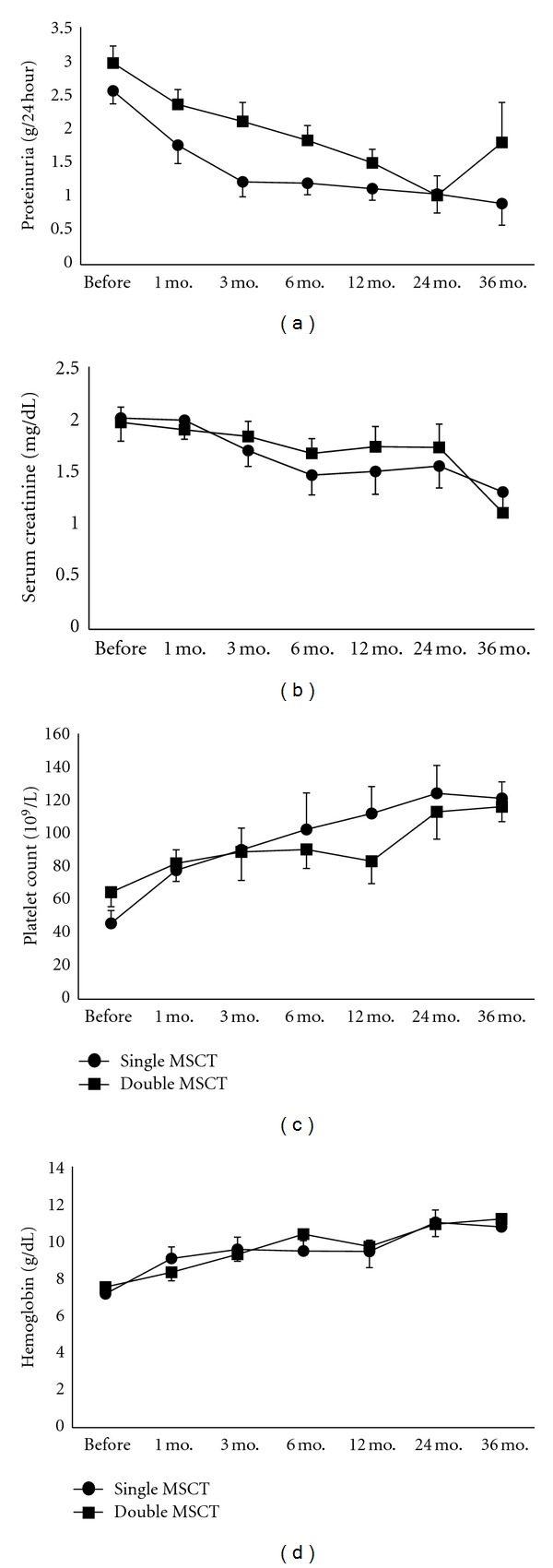
Comparisons of proteinuria (a), serum creatinine (b), platelet count (c), and hemoglobin level (d) between patients given single or double MSCs transplantations, by repeated measures of ANOVA. Values are the mean ± SEM.

Twenty-six patients (26/30, 86.7%) in single MSCT group and 24 patients (24/27, 88.9%) in double MSCT group underwent renal involvement at baseline, shown by the presence of proteinuria, or hematuria, or renal disfunction. The 24 hour proteinuria significantly declined after allogenic MSCT within each group by repeated measures ANOVA (*F* = 19.29, *P* = 0.001). However, once MSCT group demonstrated much lower levels of proteinuria compared to double MSCT group at the first 12-month visits (*F* = 5.31, *P* = 0.026, Figure 3(a)). For those who had renal disfunction at baseline, serum creatinine significantly ameliorated after MSCT within each group (*F* = 6.30, *P* = 0.003), and there was no difference between the two groups (*F* = 0.401,  *P* = 0.534, Figure 3(b)). Twelve patients (12/30, 40.0%) in single MSCT group and 13 patients (13/28, 46.4%) in double MSCT group suffered hematologic involvement at baseline; platelet counts and hemoglobin levels significant improved after allogenic MSCT in each group (*F* = 10.001, *P* = 0.000 for platelet counts, *F* = 9.237, *P* = 0.000 for hemoglobin levels); no difference was found between the two groups (*F* = 0.098, *P* = 0.760 for platelet counts, *F* = 0.015, *P* = 0.905 for hemoglobin levels, Figures 3(c) and 3(d)).

### 3.5. Adverse Events

One patient in double transplantation group underwent uncontrolled disease recurrence 6 months after MSCT due to upper respiratory tract infection. She was not responsive to conventional treatments and finally died of acute heart failure. During 4 years followup, 7 patients in single transplantation group (23.3%) and 9 patients in double transplantation group (32.1%) suffered infection events, and no statistical difference was found between the two groups. Of 7 patients in single transplantation group, 3 had upper respiratory tract infection, 3 had intestinal infection, and one had oral fungi infection. Of 9 patients in double transplantation group, 4 had upper respiratory tract infection, 2 had intestinal infection, one had herpes zoster infection, one had pneumonia, and one had pulmonary tuberculosis. All the adverse events were not considered transplantation related.

### 3.6. Maintenance Therapy

Two patients in both single (2/30; 6.7%) and double transplantation groups (2/28; 7.1%) had discontinued immunosuppressive drugs in the last followup. Dose of prednisone was tapered to 5–10 mg/day for 24 patients (24/30, 80.0%) in single MSCT group and 22 patients (22/28, 78.6%) in double MSCT group, respectively. Maintenance therapy regimen was defined as the dose of prednisone was not more than 10 mg/day, combined with the dose of immunosuppressive drug was not more than 0.4–0.6 gm/3 months of CYC, 10 mg/day of leflunomide, or 0.5 gm/day of mycophenolate mofetil. Eleven and 7 patients in single (11/30; 36.7%) and double (7/28; 25.0%) transplantation groups achieved above-mentioned maintenance therapy in the last followup. Time to reach maintenance therapy was not different between single (11.8 months, 3–24 months) and double (10.0 months, 4–15 months) transplantation groups. 

## 4. Discussion

Systemic infusions of mesenchymal stem cells have been widely used in clinical applications. However, the appropriate dose of cells for each patient is still unknown. The dose of MSCs in current studies relied to a large extent on clinical experience and lack of rigorous standards. In a phase II clinical trial for MSCs transplantation in GVHD and followed up for 5 years [19], the therapeutic dose of MSCs ranged from 0.8 million to 9.0 million per kilogram for responders and from 0.6 million to 1.9 million per kilogram for nonresponders. However, no significant correlation has been made between the dose of MSCs received and clinical outcomes. Furthermore, single, double, and repeated doses of MSCs have been administered, but with no obvious pattern to the observed outcomes. A small clinical study showed that repeated intermittent MSCs infusions, ranged from 4 to 8 times, with a 3- to 14-day interval, 10 to 20 million MSCs each time, could successfully improve signs and symptoms, as well as Th1/Th2 rebalance for 4 patients with sclerodermatous chronic GVHD [20]. Recently, Lim et al. [21] applied different dose of third-party-derived-bone marrow MSCs for two patients with GVHD (ranged from 0.5 to 2 million cells per kilogram each infusion), and the outcomes showed that a dose of one million per kilogram was as effective as that of 2 million per kilogram of recipient. Nevertheless, these case studies were insufficient to provide evidence for clinicians and larger-scale clinical trials are needed to determine the optimal cells dose for a better clinical application.

This study for the first time represents a large single-institution series of refractory SLE patients receiving single or double MSCs transplantations. We found a considerable improvement in disease remission for patients transplanted single and double allogenic MSCs. In previous studies, we have proposed that single allogenic MSCs transplantation ameliorated disease phenotype in SLE mice and humans [13]. Additionally, multiple infusions of allogenic UC MSCs, at 18, 19, and 20 weeks, seemed to significantly ameliorate lupus nephritis in MRL/*lpr* mice, compared to single transplantation [15], our animal and clinical data suggest that there may exist disparity between lupus mice and humans.

Although the routes of administration are different between diseases, such as intraportal injection for liver cirrhosis [22] and intrathecal injection for multiple sclerosis or amyotrophic lateral sclerosis [23], intravenous infusion is intensively recommended and applied for most type of diseases [24, 25]. In the present study, we focused on comparing the difference between single and multiple transplantations of allogenic MSCs intravenously, with each dose of one million cells per kilogram of body weight. The dose of infused MSCs for each transplantation was based on the previous successful treatment with the same dose in refractory SLE patients and lupus models [12–14]. Additionally, the current consensus report of the International MSCT Study Group has preferred a dose of 1-2 million MSCs per kilogram for a single intravenous infusion [26]. Based on our previous studies and current reports [15, 27], we chose one-week interval between two times of MSCs transplantation for patients. The current data revealed an optimal dose of infused allogenic MSCs for SLE patients. However, whether this is the case in other disorders still needs further investigations.

Most enrolled patients were unresponsive to CYC treatment before MSCT (for at least 6 months), the low dose of CYC given 4 days before MSCs infusion to each patient was used to inhibit active lymphocytes responses but not to treat disease. So we do not think the same pretreatment regimens before MSCT in both groups would influence the clinical response between once and double MSCT. Furthermore, the dose of CYC in the present study is much lower than that used in hematopoietic stem cells transplantation (total 30 mg/kg versus 200 mg/kg), and our previous animal studies had demonstrated that the addition of CYC before MSCT could not enhance clinical efficacy in MRL/*lpr* lupus mice [28]. Moreover, allogenic MSCT could act more effective than CYC in treating MRL/*lpr* lupus mice [13]. Recently, we have compared the clinical efficacy between patients given and not given CYC for pretreatment at baseline, and the results showed no difference between the two groups (unpublished data). So patients' clinical response was not resulted from CYC pretreatment.

The role of transplanted MSCs *in vivo* is mainly dependent on their multiple differentiation and tissue repairing, as well as extensive immune modulation [7, 29]. Although most of *in vitro* experiments showed that the immunoregulatory effect of MSCs on T cells or B cells is in a dose-dependent manner [30, 31], the reason that repeated transplantations of allogenic MSCs *in vivo* failed to enhance therapeutic efficacy in SLE patients is unclear. It is undoubted that the dose of MSCs in patients is not the more the better, and the appropriate dose of MSCs is most important for clinical treatment. There is no necessity of double transplantations for SLE patients for each therapy. More studies are needed to investigate the role of multiple infused MSCs *in vivo*.

## 5. Conclusion

This study provides evidence that single transplantation at the dose of one million MSCs per kilogram of body weight is sufficient to induce disease remission in the treatment for refractory SLE patients, and double MSCT had no enhanced effect.

## Supplementary Material

In the present study, 8 relapse events occurred in single MSCT group, at 40, 36, 24, 48, 24, 30, 12 and 18 months, respectively. Six relapse events occurred in double MSCT group, at 28, 12, 3, 24, 24 and 9 months, respectively.

## Figures and Tables

**Table 1:** Patient demographics pretransplantation.

Variable	No. of patients	
	Single MSCT	Double MSCT
	(*n* = 30)	(*n* = 28)
Age in years	30 (12–47)	33 (16–54)
Gender, *n* (F/M)	25/5	26/2
Race, *n* (Asian/others)	30/0	28/0
Disease duration (m)	62 (7–232)	92 (12–264)
MSCs source, *n*		
Bone marrow (BM)	12	9
Umbilical cord (UC)	18	19
Medium followup for survivors (m)	27 (12–48)	26 (12–40)

MSCT, mesenchymal stem cells transplantation.
