# Supplementary material for: Double Allogenic Mesenchymal Stem Cells Transplantations Could Not Enhance Therapeutic Effect Compared with Single Transplantation in Systemic Lupus Erythematosus
Source: Clin Dev Immunol. 2012 Jul 9;2012:273291. doi: 10.1155/2012/273291 (PMC3399403; doi:10.1155/2012/273291)
Supplement: Supplementary file 1 — In the present study, 8 relapse events occurred in single MSCT group, at 40, 36, 24, 48, 24, 30, 12 and 18 months, respectively. Six relapse events occurred in double MSCT group, at 28, 12, 3, 24, 24 and 9 months, respectively. [file 273291.f1.pdf]

Supplementary material:

In the present study, 8 relapse events occurred in single MSCT group, at 40, 36, 24, 48, 24, 30, 12 and 18 months, respectively. Six relapse events occurred in double MSCT group, at 28, 12, 3, 24, 24 and 9 months, respectively. The detailed information is listed below.

Single MSCT group		Double MSCT group	
Time (month)	Relapse (0)	Time (month)	Relapse (0)
40	0	40	1
36	1	28	0
36	1	40	1
36	0	12	0
30	1	3	0
48	1	24	0
48	1	24	0
24	0	36	1
48	0	18	1
30	1	36	1
24	0	24	1
30	0	24	1
24	1	24	1
24	1	24	1
12	0	24	1
18	1	24	1
18	1	18	1
18	1	18	1
18	1	18	1
18	1	9	0
18	1	18	1
18	1	18	1
18	1	18	1
18	0	18	1
18	1	12	1
18	1	12	1
18	1	12	1
18	1		
12	1		
12	1		
